# Modelling acrylamide acute neurotoxicity in zebrafish larvae

**DOI:** 10.1038/s41598-017-14460-3

**Published:** 2017-10-24

**Authors:** Eva Prats, Cristian Gómez-Canela, Shani Ben-Lulu, Tamar Ziv, Francesc Padrós, Daniel Tornero, Natàlia Garcia-Reyero, Romà Tauler, Arie Admon, Demetrio Raldúa

**Affiliations:** 1 CID-CSIC, Jordi Girona 18, 08034 Barcelona, Spain; 2 IDAEA-CSIC, Jordi Girona 18, 08034 Barcelona, Spain; 3 The Smoler Proteomics Center and the Department of Biology, Technion – Israel Institute of Technology, Haifa, Israel; 4 Fish Diseases Diagnostic Service, Facultat de Veterinària. Universitat Autònoma de Barcelona, 08190 Bellaterra (Cerdanyola del Vallès), Spain; 5 Environmental Laboratory-US Army Engineer Research and Development Center, Vicksburg, MS USA

## Abstract

Acrylamide (ACR), a type-2 alkene, may lead to a synaptopathy characterized by ataxia, skeletal muscles weakness and numbness of the extremities in exposed human and laboratory animals. Currently, only the mildly affected patients undergo complete recovery, and identification of new molecules with therapeutic bioactivity against ACR acute neurotoxicity is urgently needed. Here, we have generated a zebrafish model for ACR neurotoxicity by exposing 5 days post-fertilization zebrafish larvae to 1 mM ACR for 3 days. Our results show that zebrafish mimics most of the pathophysiological processes described in humans and mammalian models. Motor function was altered, and specific effects were found on the presynaptic nerve terminals at the neuromuscular junction level, but not on the axonal tracts or myelin sheath integrity. Transcriptional markers of proteins involved in synaptic vesicle cycle were selectively altered, and the proteomic analysis showed that ACR-adducts were formed on cysteine residues of some synaptic proteins. Finally, analysis of neurotransmitters profile showed a significant effect on cholinergic and dopaminergic systems. These data support the suitability of the developed zebrafish model for screening of molecules with therapeutic value against this toxic neuropathy.

## Introduction

Acrylamide (ACR) is a water-soluble alkene primarily used in the production of polyacrylamides. ACR polymers and copolymers are widely used in the paper and textile industries, as flocculants in the wastewater treatment and municipal drinking water, as soil conditioners, in ore processing, and in cosmetics^1^. The ACR monomers and polyacrylamides are also used as chemical grouts in tunnels, sewers and wells^2^. ACR can be also generated from heat-induced reactions between the amino group of the free amino acid asparagine and the carbonyl group of reducing sugars during baking and frying^3^. Widely consumed processed food with high levels of ACR include French fries, potato chips, tortilla chips or bread crust^1^.

Most of the evidence indicates that ACR exposure causes selective neurotoxicity in humans^4^. Reports of ACR poisoning after occupational exposure to ACR indicated major symptoms related to polyneuropathy, including ataxia, skeletal muscles weakness and numbness of the extremities^5–7^. Effects of ACR on the visual system have also been reported in humans and other species^8,9^. Early morphological studies suggested that ACR neurotoxicity was associated with central-peripheral distal axonopathy, and initial research was focused on the primacy of axon damage and on deciphering underlying mechanisms of toxicity^10^. However, evidences generated by subsequent morphometric studies on peripheral nerves suggested that axon degeneration was an epiphenomenon specifically associated to long-term, low-dose exposure^4,11^. Finally, studies by the LoPachin group demonstrated that the molecular initiating event of ACR neurotoxicity was the disruption of presynaptic vesicle cycling by selectively forming adducts with thiolate sites located on proteins specifically involved in vesicle docking (synaptotagmin, synaptophysin, and syntaxin), vesicle priming (complexin-2), SNARE core dissolution (N-ethylmaleimide sensitive factor), endocytosis (clathrin), neurotransmitter re-uptake (membrane dopamine transporter) and vesicular storage (vesicular monoamine transporter) at the nerve terminals^4^.

Currently, medical treatment of ACR neurotoxicity in humans is symptomatic, and only the mildly affected patients undergo complete recovery^12^. Different animal models for acute ACR neurotoxicity have been generated in mammalian species including rats, mice, guinea-pig, rabbits, dogs, cats and primates^13^, although most of the studies aiming to identify neuroprotective compounds against this toxic synaptopathy have used the rat model^14–16^. However, models built in rodents are not suitable for *in vivo* high-throughput screening of chemical libraries. Zebrafish (*Danio rerio*) is a vertebrate model increasingly used in biomedical research, including human toxicology studies^17–19^. One key advantage of zebrafish embryos/larvae over other vertebrate models for drug discovery is their suitability for *in vivo* high-throughput screening of chemical libraries for pharmacological and/or toxicological effects. In this context, zebrafish has been proposed as an intermediate step between cell-based assays and mammalian (and ultimately human) testing. Furthermore, zebrafish is an excellent organism for modelling human neuropathological processes, as this animal species exhibits a similar overall nervous system organization to humans and similar neurotransmitter systems, including glutaminergic, cholinergic, serotonergic, dopaminergic, adrenergic, histaminergic, GABAergic, and histaminergic^20–23^. However, any animal model suitable to be used in the identification of new drugs for treatment of ACR acute neurotoxicity should recapitulate the most relevant pathophysiological mechanisms in human^24^, and currently no data are available about the sensitivity of zebrafish larvae to develop synaptotoxicity by acute exposure to ACR.

In this study, we developed and characterized a zebrafish model for ACR acute neurotoxicity by waterborne exposure of 5 days post-fertilization (dpf) zebrafish larvae to ACR for 3 days. Pathophysiological processes involved in the development of the neuropathy in zebrafish have been characterized at different levels of organization, from the whole organism level to molecular level. Our result support that the zebrafish model for ACR acute neurotoxicity mimics most of the aspects of this process in mammals, including the formation of ACR-adducts in some proteins related with the synaptic vesicle cycling and the presence of specific effects on nerve terminals and neurotransmitter profile, indicating that zebrafish is a suitable model for screening of molecules with therapeutic value to treat this toxic neuropathy.

## Results and Discussion

### Systemic toxicity

In order to build a zebrafish model for acute ACR neurotoxicity, 5 dpf larvae were exposed for 3 days to different ACR concentrations in water. Although zebrafish is used at unlicensed stages (embryos and early larvae) for developmental neurotoxicity studies, we decided to select 5 dpf larvae for the development of the model because, while it is still suitable for high-throughput screening of chemical libraries, the potential confounding factor of neurodevelopmental processes is strongly reduced at this developmental stage. In fact, by 5 dpf neuronal proliferation is limited to only a few particular regions, with most regions of the brain comprised of post-mitotic neurons with well-elaborated neuronal arbors^25,26^. Moreover, complex behaviours such as responses to visual and acoustic/vibrational stimuli are only apparent from 5 dpf onwards^27^.

In order to select a static or semi-static exposure system for our study, a time-course analysis of the ACR stability was performed. ACR measured values were very close to the nominal values, and the concentration remained stable in our conditions for more than 5 days (Table S3). Thus, a 72h-static exposure procedure was selected for the development of the model.

The next step was to identify the optimal ACR concentration for building the model. The preferred concentration should be high enough to maximize the chance of detecting a neurotoxic effect, but not so high to induce systemic toxicity, an important confounding factor^28^. In mammalian toxicology this concentration at the threshold of the systemic toxicity is known as Maximum Tolerated Dose (MTD), and this concept has been translated to fish toxicology as Maximum Tolerated Concentration (MTC)^29^. Therefore, 5 dpf zebrafish larvae were exposed for 72 h to different ACR concentrations and the lethality and effects on gross morphology were recorded. Whereas the 50% lethal concentration (72h-LC50) was estimated 2.35 ± 0.02 mM ACR, the non-observed effect concentration (NOEC) for lethality was 1.50 mM ACR (Supplemental Fig. S1). The effects on gross morphology were then evaluated using ACR concentrations under the NOEC for lethality. No effects on gross morphology were found at ACR concentrations of 1 mM or below (Supplemental Fig. S2). Moreover, the further morphometric analysis performed on larvae exposed to 1 mM ACR (n = 32) showed no differences in body length with the control larvae (P = 0.753, one-way ANOVA with Dunnett’s multiple comparison test; Supplemental Fig. S2). Finally, as liver has been reported to be an important target for ACR systemic toxicity^30^, the potential adverse effect of 1 mM ACR exposure on the liver of the exposed larvae was analysed at histological level. Results from the histopathological analysis showed that liver histology was well preserved in ACR-treated larvae (Supplemental Fig. S2). Therefore, MTC for systemic toxicity of ACR in larvae exposed from 5 dpf to 8 dpf was 1 mM ACR, and this concentration was the highest concentration tested for the neurotoxicity analysis.

### Effects on motor function

ACR neurotoxicity in humans and mammalian animal models is characterized by an impairment in the motor function^4^. Thus, motor function was analysed in control and ACR-treated larvae by using a battery of test including basal locomotor activity (BLA), visual motor response (VMR), acoustic/vibrational motor response and the touch-evoked escape response.

**Figure 1:**
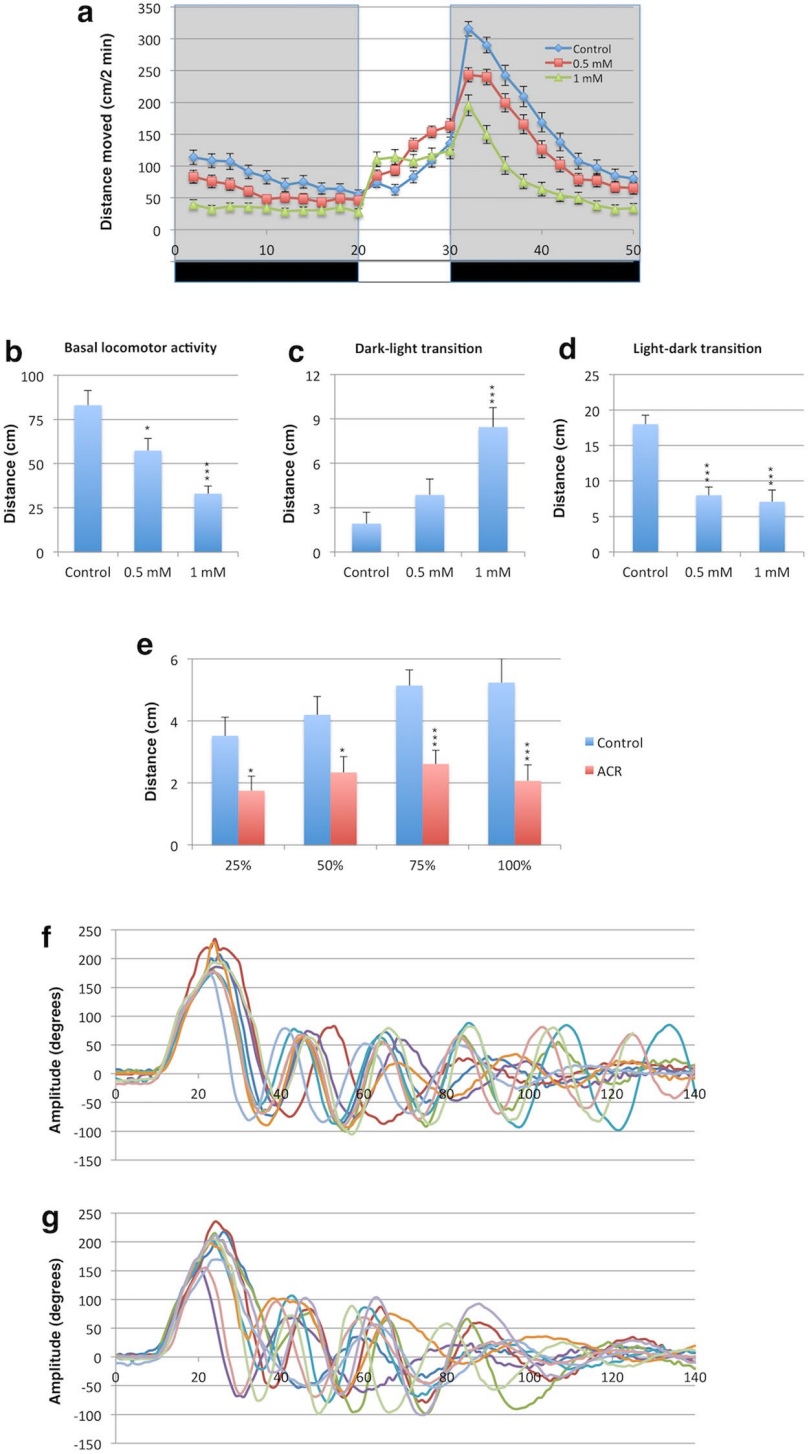
Motor response is strongly impaired by acrylamide (ACR) in zebrafish larvae. **(a)** Locomotor activity of 8 days post-fertilization zebrafish larvae control (n = 78) and exposed to 0.5 mM ACR (n = 79) and 1 mM ACR (n = 77) during a 20 min dark period followed by a 10 min light period and then a second cycle of 20 min of darkness. Data from 3 independent experiments. **(b)** Basal locomotor activity (BLA), defined as the distance moved by the larvae during the first period of 20 min in the dark, was significantly reduced by ACR. Statistical analysis performed using one-way ANOVA with Dunnett’s multiple comparison test;; *P < 0.05, ***P < 0.00001; Results represent mean ± sem. **(c)** 1 mM ACR, but not 0.5 mM ACR, induced a period of hyperactivity in the dark to light transition (the difference in activity between the 2 first min with light and the last 2 min of the first dark period is represented). Statistical analysis performed using one-way ANOVA with Dunnett’s multiple comparison test; ***P < 0.00001; Results represent mean ± sem. **(d)** Visual motor response (VMR), the hyperactivity period evoked by a sudden reduction in light intensity, is strongly reduced in larvae exposed to 0.5 and 1 mM ACR. Statistical analysis performed using one-way ANOVA with Dunnett’s multiple comparison test; ***P < 0.00001; Results represent mean ± sem. (**e**) Larvae exhibiting a total abolition of the VMR after 1 mM ACR exposure exhibit a significant reduction in the acoustic/vibrational motor response evoked by a solenoid at four different intensities. Statistical analysis using Student’s t-test, *P < 0.05, ***P < 0.001; Data from 20 control and 22 ACR-treated larva from 5 independent experiments); Results represent mean ± sem. **(f,g)** Kinematic of the touch-evoked escape response is altered in zebrafish larvae exposed to 1 mM acrylamide. Representative kinematic traces of the touch-evoked escape response of control **(f)** and ACR-treated **(g)** larvae. For each condition eight representative traces are shown from the first 140 ms of the escape response. Each trace is from a different larvae. The curvature of the body is represented in degrees, with 0 indicating straight body.

BLA, defined as the distance moved by the larvae in 20 min, was significantly reduced in larvae exposed to 0.5 mM ACR (*P* < 0.05) and 1 mM ACR (*P* < 0.0001) compared to the control (Fig. 1a,b).

Sudden increments or decrements in light intensity elicit acute locomotor response in zebrafish larvae^25^. Therefore, the effects of sharp changes in light intensity on ACR-treated larvae were evaluated. When the locomotor response of larvae to a sudden increase in light intensity was analysed during the dark to light transition period, a significant increase (*P* < 0.0001) in locomotor activity was found only in larvae exposed to 1.0 mM ACR compared to the controls (Fig. 1a,c). VMR, a transient period of hyperactivity exhibited by zebrafish larvae in response to sudden decrease in light intensity^31^, was evaluated by tracking larvae in response to light-dark transitions. Figure 1a,d shows a clear decrease in the VMR in larvae exposed to 0.5 mM and 1 mM ACR. As 1 mM ACR, but not 0.5 mM ACR, was able to impair the motor response of larvae to light and dark flash stimuli we selected 1 mM ACR for the development of the acute neurotoxicity model. The locomotor response induced by sudden increments or decrements in light intensity integrates in fact sensory and motor function. By 5 dpf, larvae transmit the information about increments and decrements in light intensity through the retinal ON and OFF channels, two differentiated circuits relaying visual signals from photoreceptors to bipolar and ganglion cells and ultimately to higher visual centers^32^. The different response of ACR-treated larvae to increments and decrements in light intensity strongly suggest specific effects on the retinal function. Different effects on the visual system have been reported after accidental ACR-poisoning in humans and cows^8,9^. Moreover, visual impairment has been reported in different mammalian models for ACR neurotoxicity, including primates and rodents^33–36^. Interestingly, it has been demonstrated that impairment of the presynaptic vesicle cycling, the ACR mode of action in mammals, results in an abnormal response to sudden changes in light intensity in the zebrafish mutant *no optokinetic response c* (*nrc*), exhibiting a premature stop codon in *synaptojanin 1* gene^32^. Thus, the observed effects in ACR-treated larvae are consistent with the reported effects on the visual system in mammalian models.

When the distribution of VMR in individual larvae was analysed, the main effect observed after ACR-treatment was a clear increase in the frequency of larvae exhibiting a negative or very low VMR response to the sudden decrease in light intensity (Supplemental Fig. S3). For other ACR-treated larvae, however, the VMR values were in the same range than in control larvae. As the zebrafish acute ACR neurotoxicity model needs to exhibit a clear neurobehavioral phenotype, we decide to selected only those ACR-exposed larvae with VMR values below the percentile 10th of the control values for building the model. Larvae collected for further behavioural, histopathological, neurochemical and transcriptomic analyses were selected using that criteria (Supplemental Fig. S4).

We analysed also the effect of ACR on the acoustic/vibrational motor response by tapping the plate with a solenoid at four different intensities. Acoustic/vibrational stimuli generated by tapping a plate are multimodal, and response is triggered by the differential acceleration of the otoliths of the inner ear compared to the body^25^. ACR-exposed larvae exhibited a significant moderate decrease (44–60%) in the motor response evoked by the acoustic/vibrational stimuli (Fig. 1e). Interestingly, one of the early signs of ACR reported in occupationally exposed workers and experimental animals was the impairment of the vibration sensation^37,38^.

Finally, we analysed the effect of ACR on the kinematic of the touch-evoked escape response, a highly stereotyped complex behaviour constituted by three sequential modules: (1) a very fast and large C-bend followed by (2) a high amplitude counterbend and (3) a bout of fast swimming oriented away from the stimulus. The number of swim cycles performed by the larvae during the last module is highly variable. Whereas kinematic analysis of control larvae exhibited the stereotyped profile, ACR-treated larvae exhibited some abnormalities in the escape response (Fig. 1f,g and Table 1). Thus, whereas ACR induced only a mild effect on the C-bend, the fast swimming module was specifically targeted by this neurotoxicant (Table 1). Although no differences were found in the amplitude, the frequency of the swimming cycles and the total distance moved in 100 ms during the fast swimming module were significantly reduced (Table 1). The decrease in the frequency of the fast swimming module could also explain, at least partially, the reduction in the distance moved observed in the treated larvae after the acoustic/vibrational stimuli. However, the total abolition in the motor response after a sudden decrease in light intensity found in ACR-treated larvae supports the hypothesis that ACR targets specific neuronal circuits at the retina level.

**Table 1:** Kinematic parameters of the touch evoked escape response of control and ACR-treated zebrafish larvae.

	***Control***	***1 mM ACR***	***P***
*Duration of C-bend (ms)*	13.45 ± 0.40	12.17 ± 0.58	0.043
*Amplitude of C-bend (degrees)*	183.60 ± 4.55	166.85 ± 5.53	0.014
*Frequency fast swimming module(Hz)*	39.79 ± 1.93	32.47 ± 1.27	0.0011
*Amplitude during fast swimming module (degrees)*	138.99 ± 6.90	125.89 ± 11.10	0.1635
*Number of full cycles in 100 ms*	3.10 ± 0.23	2.33 ± 0.15	0.0034
*Distance moved in 100 ms (mm)*	7.51 ± 0.73	5.09 ± 0.43	0.0047

Results represent mean ± sem. Statistical analysis performed using Student’s t-test. Data from 2 independent experiments.

### Histopathological analysis

**Figure 2:**
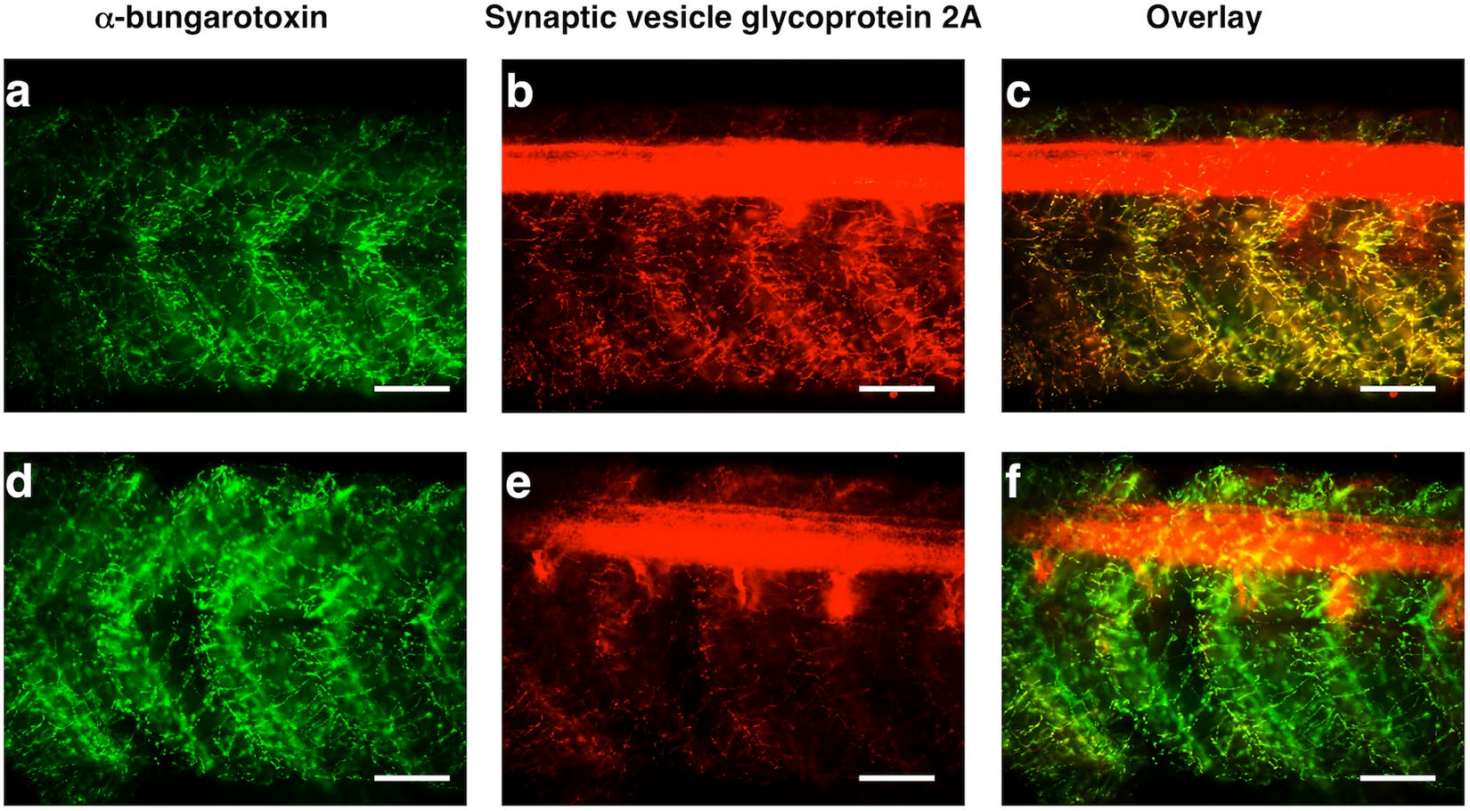
ACR reduces presynaptic nerve terminals in zebrafish larvae. At the neuromuscular junctions (NMJ) of the trunk, ACR-exposed larvae exhibit an strong reduction in the labelling of synaptic vesicle glycoprotein 2a (marker of synaptic terminals of the spinal motor neurons), whereas the α-bungarotoxin labelling (post-synaptic marker at the NMJ) labelling remains unaltered. Detail of the trunk, in lateral view, of control (**a**–**c**) and ACR-treated (**d**–**f**) larvae after co-labelling with α -bungarotoxin Alexa Fluor 488 conjugate (**a,d**) and SV2 antibody. The co-localization of the pre-synaptic and post-synaptic markers of NMJ is also showed (**c,f**). Scale bar: 100 μm.

Whereas the histopathological effects of chronic exposure to ACR on the peripheral nervous system include a primary axonal degeneration with a secondary demyelination, the acute effects are more restricted to the nerve terminals^4,13^. Synaptic terminal degeneration at the NMJs, however, has been reported after both acute and chronicle exposure^39,40^. First of all, histopathological alterations in ACR-exposed larvae where evaluated in semithin sections stained with toluidine. No differences where found in the retinal architecture and CNS between the control and treated larvae by using this approach (Supplemental Fig. S5). However, although toluidine blue staining allows to detect extended severe histopathological alterations across the nervous system^20^, it is probably not the most sensitive staining for identifying subtle changes on the retina or nervous system. Thus, whereas toluidine blue allowed to detect a severe damage in the retina and CNS in a zebrafish model for severe acute organophosphorus poisoning (OPP), the same staining failed to identify any histopathological effect in a zebrafish model for mild OPP exhibiting altered BLA and VMR^20^. We decided then to use a whole-mount immunofluorescence to assess specifically potential effects of ACR on axonal tracts or myelin sheaths at the peripheral nervous system. No effects of ACR were found in the morphology or in the intensity of the labelling of axonal tracts and the myelin sheaths by using the primary antibodies 3A10 and anti-mbp, respectively (Supplemental Fig. S6). However, when the effect of ACR on the neuromuscular junctions (NMJs) was analysed, a consistent decrease in the labelling synaptic terminals of the spinal motor neurons, was found (Fig. 2), with no effect at the post-synaptic side. Independently of the mechanism (nerve terminal degeneration, down-regulated protein synthesis, increased degradation) behind the observed reduction in SV2 immunofluorescence, this protein seems to be involved in the synaptic docking and priming in the synaptic terminals, and the impairment in the levels of this protein results in altered neurotransmission^41^.

Thus, results of the histopathological assessment in the zebrafish model for ACR acute neurotoxicity were consistent with the reported effects in mammalian models, with effects at the nerve terminal level, but not on the axons or myelin sheaths.

### Effects on transcriptional level

**Figure 3:**
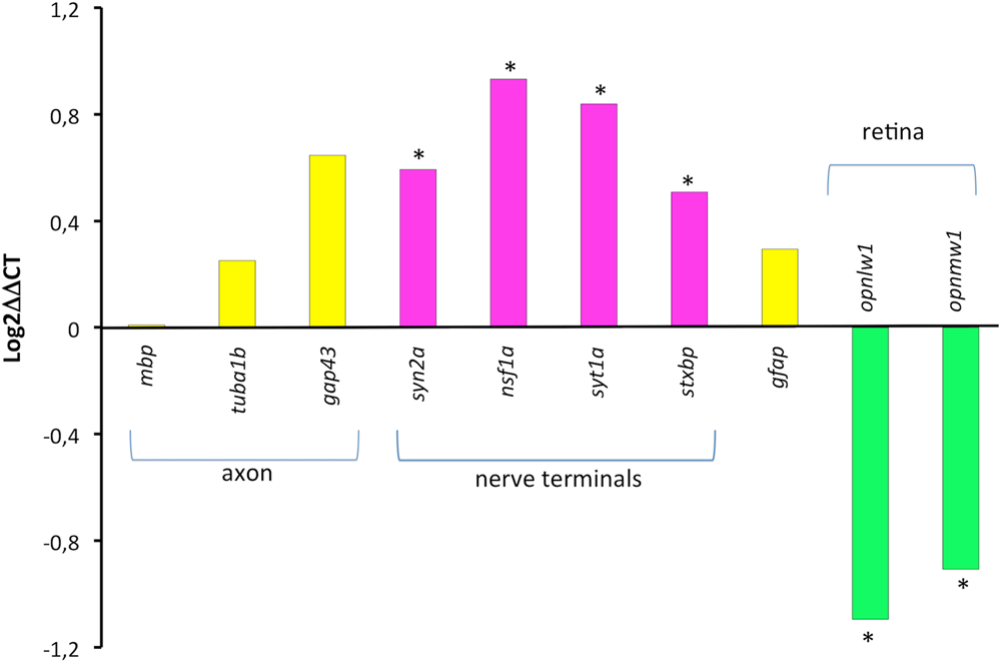
ACR exposure induces a significant change en the expression of transcripts related with the synaptic vesicle cycling and visual function.

In order to characterize better the ACR targets in the CNS of the zebrafish larvae, the expression of ten genes involved in synaptic vesicle cycling, axonal integrity, myelin sheaths, reactive astrocytes and retinal photoreceptors was analysed. Consistently with the histopathological results, no differences were found in the expression of genes encoding the axonal proteins α-tubulin and GAP-43 nor in the myelin basic protein (Fig. 3). There were also no differences in the expression of GFAP, the major intermediate filament protein of astrocytes commonly used as neurotoxicity marker. However, ACR-exposed larvae exhibited a significant up-regulation in the expression of *syn2a*, *nsf1a*, *syt1a* and *stxba*, all genes involved in the synaptic vesicle cycling. This result, consistent with the histopathological findings, strongly suggest that, as in mammals, nerve terminals, and not axons or myelin sheaths, are the primary target of ACR in zebrafish larvae. Finally, consistent with the altered response to sudden increases and decreases in light intensity found in ACR-exposed larvae, a significant down-regulation was found in two opsin genes, expressed in the photoreceptors of the retina. An effect in the visual system and opsin expression was reported in the zebrafish model for mild OPP^20^.

### Effects of ACR on the fish proteome

Synapses are highly sophisticated computational units built from proteomes containing in excess of 1,000 proteins that regulate behaviour repertoire^42^, and loss of function of some of these proteins by genetic or toxicological factors results in many different neurological disorders. The current view is that ACR induce synaptotoxicity in mammals and that the molecular initiating event is the formation of ACR-adducts in cysteine residues involved in the synaptic vesicle cycle at the nerve terminal, resulting in altered function of the modified proteins and, finally, in altered neurotransmission^4^. In order to determine if ACR altered the expression of proteins involved in synaptic vesicle cycle in the nerve terminal of zebrafish, the proteome of control and exposed larvae was compared. It is important to consider that, although most of the proteomic studies in mammals on the effect of ACR on nerve terminals use isolated synaptosomes, this approach is impracticable in zebrafish larvae. Whereas isolation of synaptosome require 0.7–1 g of neural tissue^42^, the wet weight of one 8 dpf zebrafish larva is about 0.28 mg, which means that for a single biological replicate the brain of thousand of larvae should be sacrificed and dissected.

Five pools of larvae from each group were proteolyzed and analysed by capillary chromatography and tandem mass spectrometry (μLC-MS/MS). A total of 4,498 proteins were identified in at least 3 of the samples and with at least 2 different tryptic peptides (Supplementary Dataset 1). The levels of 88 proteins were differentially affected with at least 2-fold difference, 48 down-regulated and 38 up-regulated, indicating that the ACR exposure had only a limited effect at the whole proteome level (Supplementary Figs S7–S9 and Supplementary Dataset 2). Interestingly, although proteome analysis was performed on the whole larvae, instead of using synaptosomes, the most relevant proteins involved in synaptic vesicle cycle (neurotransmitter uptake, synaptic vesicle translocation, docking, priming, fusion and fission) were detected (Supplementary Dataset 3). Nevertheless, our results show that ACR had no effect in the expression of the most relevant proteins involved in synaptic vesicle cycle (Supplementary Dataset 2). Normal levels of synaptosomal proteins were also found in rats exposed to ACR^43^, a result suggesting that changes in protein expression are not involved the observed neurotoxic effects.

**Figure 4:**
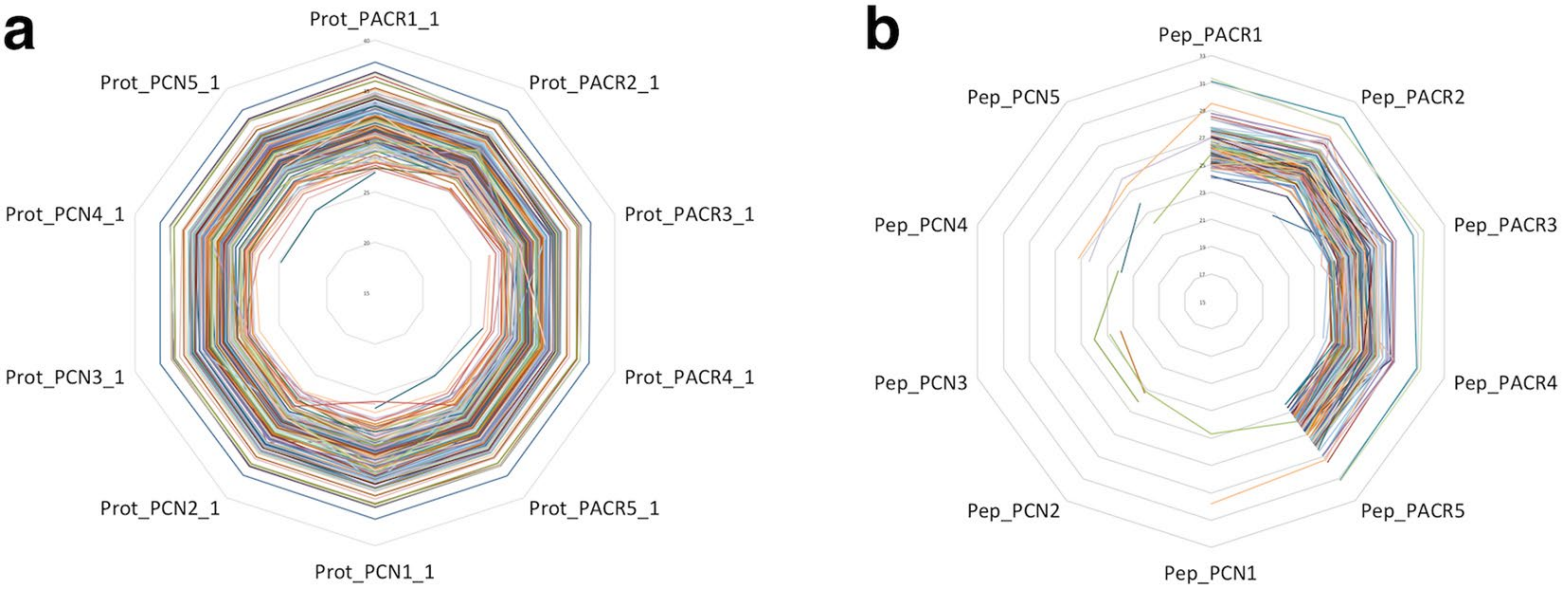
Proteins containing ACR-modified cysteine residues in at least 3 treated samples. (**a**) Expression levels of the 138 proteins containing ACR-modified cysteine residues; (**b**) Log-2 intensities of the peptides with ACR –adducts in cysteine residues. PCN: control pool; PACR: pool ACR.

In addition, when the presence of covalent acrylamide adducts was analysed in control and ACR-exposed larvae, 138 proteins were detected with modifications on specific cysteine residues in at least 3 of the ACR-pools (Fig. 4 and Supplementary Dataset 4), and in most of these, the expression levels of the proteins were not changed as a result of the modification. The most significantly propionamide-modified group of proteins were the crystallins, especially βb1-crystallin (Q6DGY4). This protein is expressed not only in the ocular lens but also in the brain. Mutation in the crystalline encoding genes are causative for various forms of congenital or juvenile cataracts, and *crybb1* is causative of congenital or juvenile eye disorders and is also involved in neurological disorders including schizophrenia^44^. Interestingly, recent evidences from schizophrenia studies have demonstrated that key proteins involved in presynaptic release mechanisms are dysregulated in this disorder^45^. Moreover, βb1-crystallin is also involved in fear and stress-associated responses, with its repression reducing anxiety-like behavior^44,46^. The potential involvement of the ACR-adducts in crystalline proteins in the altered VMR observed in zebrafish larvae will remain to be determined.

Interestingly, some proteins specifically involved in the synaptic vesicle cycle, were also modified by ACR. Thus, we have found that ACR forms adduct with Cyst90 of complexin 2 (E7FBR8), a cytosolic protein that preferentially binds to syntaxin within the SNARE complex, promoting neuronal exocytosis. ACR adducts also have been found at Cyst417 of synapsyn II, a phosphoprotein binding synaptic vesicles to components of the cytoskeleton which prevents them from migrating to the presynaptic membrane and release their neurotransmitters^45^. Finally, ACR formed adducts at Cyst142 of vesicle-associated membrane protein (VAMP)/associated protein A (Q6P0D4). Whereas VAMP/synaptobrevin forms together with SNAP-25 and syntaxin the presynaptic SNARE complex, VAPA interacts with VAMP and is necessary for vesicular neurotransmission^47^.

Among the proteins modified in the zebrafish larvae we found ubiquitin carboxyl terminal hydrolase (Q6YI49), forming adducts with Cyst71. This protein, critical to normal ubiquitination and protein degradation, has been also reported to be modified in rat synaptosome^48^. Moreover, glyceraldehyde-3-phosphate dehydrogenase (GAPDH), forming adducts with Cyst150 in zebrafish, has been reported to be inhibited by ACR in humans after forming adducts with Cyst152. Finally, other proteins reported to be modified in rat synaptosomes, including aspartate amino transferase (Q7ZUW8), isocitrate dehydrogenase (Q7ZUP6) and ubiquinol-cytochrome c reductase (Q6PBH6), were also found to be modified in the whole zebrafish larvae extracts^48^.

Whereas most of the proteins modified by ACR in this study and other in mammals are not related specific of the nervous system, the specific synaptotoxicity induced by ACR has been explained by the fact that in nerve terminals the rate of adduction formation exceeds the rate of removal by protein turnover, and consequently ACR-modified protein will be specifically accumulate at this location^4^.

### Changes in the neurochemical profile

**Figure 5:**
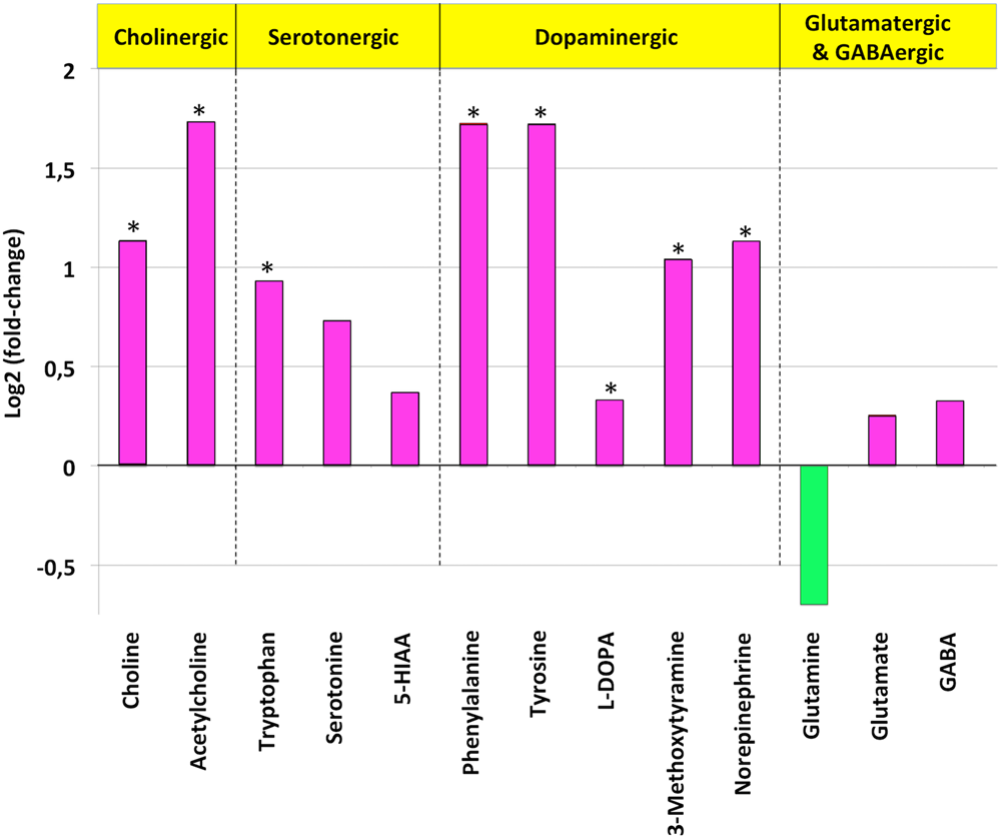
Changes in the profile of 13 neurochemicals (neurotransmitters, precursors, metabolites) in the zebrafish model for ACR acute neurotoxicity. Values are represented as log2 of fold change to control. Statistical analysis performed using Student’s t-test, *P < 0.05. Data from 2 independent experiments.

It has been demonstrated that ACR exposure impairs neurotransmission and this effect might be related to the impaired neurotransmitter release resulting from the disruption of presynaptic vesicle cycling^43,49^. In order to assess if neurotransmission was also altered by ACR in zebrafish, 26 neurochemicals, including neurotransmitters, precursors, metabolites and neuromodulators, were analysed in pools of 5 larvae from the control (n = 5) and treated (n = 6) groups (2 independent experiments). The heat map of hierarchical cluster analysis (Supplementary Figure S10) showed clear differences in the neurochemical profile, with increased levels in most of the measured neurochemicals in ACR-treated larvae. Changes in the neurochemical profiles have been recently reported in zebrafish larvae exposed to different neurotoxicants during early development^50,51^. However, results from neurochemical analyses performed on whole embryos or larvae should be taken with caution, as many of the analyzed chemicals are expressed also in non-neural tissue. When the main neurotransmitter systems were analysed (Fig. 5 and Table S4), a significant effect of ACR on the cholinergic and dopaminergic systems was found. Thus, although the dopamine levels were below the detection limit of the analytical method, the levels of the dopamine precursors (phenylalanine, tyrosine and L-DOPA) and metabolites (3-methoxytyramine and the neurotransmitter norepinephrine) were significantly increased in ACR-exposed larvae. Levels of acetylcholine and its precursor/metabolite choline were also increased in the treated larvae. Whereas the average levels of serotonin and its metabolite 5-HIAA were also higher than the control values, these differences were not statistically significant. The altered neurochemical profile in ACR-exposed larvae is consistent with the reported changes in the levels of DA, serotonin and 5-HIAA in different regions of the rat brain^52–54^.

## Conclusions

A new ACR acute neurotoxicity model have been developed and characterized in zebrafish larvae. This model mimics most of the aspects of this process in mammals, such as impaired motor function and specific effects at the nerve terminals, including altered neurotransmission. Moreover, ACR formed adducts in cysteine residues of some proteins related with the synaptic vesicle cycling in our model, which is considered as the molecular initiating event in mammalian models. All these data support the suitability of the developed model for screening of molecules with therapeutic value to treat this toxic neuropathy.

## Methods

### Fish husbandry and larvae production

Adult wild-type zebrafish were maintained in fish water [reverse-osmosis purified water containing 90 µg/ml of Instant Ocean (Aquarium Systems, Sarrebourg, France) and 0.58 mM CaSO_4_ .2H_2_O] at 28 ± 1 °C in the Research and Development Centre of the Spanish Research Council (CID-CSIC) facilities under standard conditions. Embryos were obtained by natural mating and maintained in fish water at 28.5 °C on a 12L:12D photoperiod. Larvae were not fed during the experimental period. All procedures were approved by the Institutional Animal Care and Use Committees at the CID-CSIC and conducted in accordance with the institutional guidelines under a license from the local government (agreement number 9027).

### Stability of ACR in water determination

Stability of ACR in water under exposure conditions was tested by LC-MS/MS (see Supplementary Methods for additional details).

### Experimental protocol

ACR (CAS#79-06-1, ≥99% purity) was purchased from Sigma-Aldrich (A9099; St. Louis, MO). On the day of the experiment, a fresh stock solution (500 mM ACR) was prepared directly in fish water, and then exposure solutions were prepared by diluting the stock in fish water.

For the development of the model zebrafish larvae were transferred to 48-well plates (1 larvae per well) at 5 dpf and exposed for 72 h to 0.5–3.0 mM of ACR (Sigma-Aldrich, St. Louis, MO), at 28.5 °C and a 12L:12D photoperiod. Control larvae were maintained in fish water under identical conditions.

### Concentration-response analysis for lethality

LC50s was obtained by fitting responses relative to control treatments (R) to the nonlinear allosteric decay regression model (see Supplementary Methods for additional details).

### Gross morphological analyses

Gross morphology of the control and ACR-treated larvae was analysed with a Nikon SMZ1500 dissecting microscope. Images were acquired with a Nikon Digital Sight DSRi1 camera and NIS Elements AR software (version 3.0, NIKON Instruments INC, New York, USA) and saved as 1280 × 1024 tagged image file format (TIFF).

### Behavioural analysis

BLA and VMR analyses of 8 dpf zebrafish larvae were performed using a DanioVision system running an Ethovision XT 11 software (Noldus, Wageningen, the Netherlands), essentially as described by Faria *et al*. (2015) (see Supplementary Methods for additional details).

Vibrational-evoked escape response was analysed by using the DanioVision Tapping Device DVTD-0010, working as a startle stimulus for the zebrafish larvae, in combination with EthoVision XT 9. The escape response evoked by stimuli of different intensity (25%, 50%, 75% and 100% of maximal intensity) were analysed in the control and ACR-exposed larvae (20–24 larvae per condition, from two independent experiments). Videos of 2 min were acquired at 60 fps in dark conditions. Distance moved in the 1 sec period before and after triggering the tapping stimulus was calculated with Ethovision software. The acoustic/vibrational motor response was calculated as the difference in the distance moved 1 sec after and before the stimuli.

For the touch-evoked escape response, startle responses were evoked in 8 dpf control and ACR-exposed larvae (20–27 larvae per condition from three independent experiments) by a light-touch stimulus applied to the rostral head skin using a glass capillary injection needle. Video recordings were made with a high-speed Photron Fastcam MinI UX100 camera (Photron USA Inc., San Diego, CA, USA) and kinematic analysis was performed using the Flote software package (see Supplementary Methods for additional details).

### Histopathological analysis

Semithin sections of control and treated larvae were stained with toluidine blue, essentially as described by Faria *et al*. (2015) (see Supplementary Methods for additional details).

### Immunohistochemistry and neuromuscular junctions imaging

For whole mount immunohistochemistry, larvae were fixed in 4% paraformaldehyde (PFA) in phosphate buffer saline (PBS) overnight at 4 °C and depigmented (3% H_2_O_2_ and 1% KOH in water, 40 min). Permeabilization was achieved by successive incubations in dH_2_O for 5 min, acetone (7 min at −20 °C), and dH_2_O for 5 min, followed by a final 40 min incubation in 2 mg/mL collagenase (Sigma-Aldrich, C9891). Larvae were blocked for 2 h in blocking buffer (4% goat serum, 1% BSA, 1% DMSO, 0.8% Triton X-100, and 0.1% Tween-20 in PBS, pH 7.4) and incubated overnight at 4 °C in either rabbit polyclonal anti-mbp (custom produced) at 1:1000 and mouse monoclonal antibody and 3A10 (DSHB, University of Iowa, Iowa) at 1:200. The polyclonal antibody anti-mbp, raised against the zebrafish Mbp peptide CSRSRSPPKRWSTIF (Peptide 2.0 Inc., VA, U.S.A.) at CID-CSIC (Barcelona, Spain), was used to detect oligodendrocytes and Schwann cells^55^, whereas 3A10 primary antibody was used to detect axonal tracts. Larvae were then washed for 3 h and incubated in the corresponding secondary antibody at room temperature for 2 h. The secondary antibodies used were goat anti-rabbit IgG (H + L) Alexa Fluor 555 and goat anti-mouse IgG (H + L) Alexa Fluor 488 IgG (1:300; Molecular Probes).

For the neuromuscular junctions (NMJs) analysis, larvae were fixed in 4% PFA overnight at 4 °C, permeabilized (acetone cracking and collagenase treatment, as described above), blocked and incubated overnight at 4 °C with the mouse monoclonal antibody SV-2 (D1:200; SHB, University of Iowa, Iowa) and Alexa Fluor 488-α-bungarotoxin (1:100; Molecular Probes). Larvae were then washed for 3 h and incubated in goat anti-mouse IgG (H + L) Alexa Fluor 555 IgG (1:300; Molecular Probes). SV2 antibody was used as presynaptic marker, whereas α-bungarotoxin was used as post-synaptic nAChR marker at the NMJ level.

Larvae were mounted and imaged using a Nikon Eclipse 90i (Nikon, Champigny sur Marne, France) microscope fitted with Nikon Intensilight C-HGFI unit.

### RNA preparation and qRT-PCR analysis

Total RNA was extracted from pools of 4 larvae using Trizol Reagent (Invitrogen Life Technologies, Carlsbad, CA). RNA concentration was then measured by spectrophotometric absorption in a NanoDrop ND-8000 spectrophotometer (NanoDrop Technologies). After DNaseI treatment (Ambion, Austin, TX), total RNA was retro-transcribed to cDNA with First Strand cDNA synthesis Kit (Roche Diagnostics, Mannheim, Germany) according to manufacturer’s instructions. Real Time PCR was performed in LightCycler ® 480 Real-Time PCR System (Roche Diagnostics, Mannheim, Germany) using SYBR Green PCR Master Mix (Roche Diagnostics, Mannheim, Germany). Cycling parameters were 95 °C for 15 min followed by 45 cycles of 95 °C for 10 s and 60 °C for 30 s. For each experimental condition qPCR analyses were performed from two independent experiments, with 4 or 5 biological replicates on each experiment and three technical replicates for each sample. Appropriate primers for the ten selected genes (*gap43*, *gfap*, *mbpa*, *nsfa*, *opn1lw1*, *opn1mw1*, *tuba1b*, *syn2a*, *syt1a*, *sytxbp1b*) were designed using Primer Express 2.0 software (Applied Biosystems, Foster City, CA) and the Primer-Blast server (http://www.ncbi.nlm.nih.gov/tools/primer-blast; primer sequences in Table S2) and synthesized by Sigma. House-keeping gene ppia2 was selected as reference gene^56^.

Relative mRNA abundances of different genes were calculated from the second derivative maximum of their respective amplification curves (Cp, calculated by triplicates). To minimize errors on RNA quantification among different samples, Cp values for target genes (Cptg) were normalized to the average Cp values for ppia2, used as reference gene, following Eq. (1)

1 $${\rm{\Delta }}\mathrm{Cptg}={\rm{Cpppia2}}-{\rm{Cptg}}$$

Changes in mRNA abundance in samples from different treatments were calculated by the ΔΔCp method^56^, using corrected Cp values from treated and non-treated samples Eq. (2)

2 $${\rm{\Delta }}\mathrm{Cptg}={{\rm{\Delta }}\mathrm{Cptg}}_{-}{\rm{untreated}}-{{\rm{\Delta }}\mathrm{Cptg}}_{-}{\rm{treated}}$$

Fold-change ratios were derived from those values.

### Proteomics analysis

Protein fractions were extracted from pools of 8 larvae using Trizol Reagent (Invitrogen Life Technologies, Carlsbad, CA) and precipitated by 80% acetone dried for the proteomics study. The protein were extracted from the dry pellets with 9 M urea, 400 mM ammonium bicarbonate and 10 mM DTT. 20 μg protein from each sample were warmed up to 60 °C for 30 min and modified with 8.8 mM iodoacetamide in 100 mM ammonium bicarbonate (in the dark, room temperature for 30 min), diluted with water and digested in 2 M urea, 25 mM ammonium bicarbonate with modified trypsin (Promega) at a 1:50 enzyme-to-substrate ratio, overnight at 37 °C, followed by an additional trypsinization for 4 hours.

The resulting tryptic peptides were desalted using disposable C18 tips (Harvard Apparatus) dried and re-suspended in 0.1% formic acid. They were analysed by LC-MS/MS using a Q-Exactive-Plus mass spectrometer (Thermo-Fisher Scientific) fitted with a capillary HPLC (Easy nLC 1000, Thermo-Fisher Scientific). The peptides were loaded onto a homemade capillary column (about 25 cm long and 75 micron ID) packed with 3.5 m silica ReproSil-Pur C18-AQ resin (Dr. Maisch GmbH, Ammerbuch-Entringen, Germany) in solvent A (0.1% formic acid in water)^57^. The peptides were resolved with a linear gradient from 5% to 28% of solvent B (95% acetonitrile with 0.1% formic acid) during 105 minutes, followed by gradient of 15 minutes from 28 to 95% B and 15 minutes at 95% B at flow rates of 0.15 *μ*l/min. Mass spectrometry was performed in a positive ion mode (m/z 350-1800, resolution 70,000) using repetitively full MS scans, followed by higher energy collision induces dissociation (HCD, at 35 normalized collision energy) of the 10 most dominant ions (>1 charges) selected from the full MS scan. The AGC settings were 3 × 106 for the full MS and 1 × 105 for the MS/MS scans. The intensity threshold for triggering MS/MS analysis was 1 × 104. A dynamic exclusion list was enabled with exclusion duration of 20 sec.

### Proteomics Data Analysis

The mass spectrometry data was analysed using the MaxQuant software 1.5.2.8 (www.maxquant.org)^58^ fitted with the Andromeda search engine^59^, searching against the *Danio rerio* Uniprot database (of march 2017 containing 59,064 entries) with mass tolerance of 20 ppm for the precursor masses and the fragment ions. Oxidation on methionine, propionamide on cysteine, histidine and lysine were accepted as variable modification and carbamidomethyl on cysteine was accepted as fixed modifications. Minimal peptide length was set to six amino acids and a maximum of two miscleavages was allowed. Peptide and protein level false discovery rates (FDRs) were filtered to 1% using the target-decoy strategy. The identified protein table was filtered to remove the identifications from the reverse database, the common contaminants and single peptide identifications.

The data was quantified by normalized label free analysis using the same MaxQuant software (LFQ intensities), based on extracted ion currents (XICs) of peptides enabling quantitation from each LC/MS run for each peptide identified in any of experiments.

Statistical analysis of the identification and quantization results was done using Perseus software 1.5.1.6^60^. Student T-test was done with 0.05 FDR and 250 randomizations. Proteins with P value less than 0.05 and difference of at least 2 fold between the groups were labelled as differential.

### Analysis of neurochemicals by LC-MS/MS

Pools with 5 control or ACR-treated larvae were extracted by a method adapted from Gomez-Canela *et al*.^61^. Samples were spiked with 500 ng of isotope labeled solution of L-aspartic acid-^15^N (internal standard) and then, 500 μL of MeOH:H_2_O (90:10) were added to each pool and shaken. Three stainless steel beads (3 mm diameter) were placed in each sample and were homogenized using a bead mill homogenizer (TissueLyser LT, Qiagen) at 50 oscillations per min during 90 s. After this, samples were shaken at 4 °C for 20 min and then, centrifuged for 20 min at 13,000 rpm, also at 4 °C. The supernatant was filtered using 0.20 μM PTFE filters (DISMIC -13 JP, Advantec®) and stored at −80 °C until LC-MS/MS analysis. Neurotransmitters were measured using liquid chromatography connected to a triple quadrupole detector (Xevo TQD, Waters, USA) (LC-MS/MS). A Synergi Polar-RP 80 Å column (250 mm × 4.6 mm ID, particle size 4 µM, Phenomenex, Torrance, USA) was used to separate the target compounds. The mobile phase composition consisted of binary mixtures with 0.1% of formic acid in water (A) and 0.1% formic acid in MeOH (B). Gradient elution started at 95% A and 5% B in the first 2 min and increased to 30% B in 5 min. Then, gradient increased to 95% B in the next 13 min, and held for 5 min. Initial conditions to stabilize the system were attained in 5 min being 30 min the total run time. The flow rate was set at 600 µL min^−1^ and 10 µL were injected. Neurotransmitters were measured under positive electrospray ionization (ESI+). Flow injection analysis (FIA) was performed to obtain the optimum cone voltage (between 1 and 80 V) for the determination of the molecular ion and to obtain the optimum collision energies (between 1 and 50 eV) to determine at least two intense fragments. Finally, to identify each compound, acquisition was performed in selected reaction monitoring (SRM) mode using two transitions from precursor ion to product ions. More details about the chromatographic and mass spectral conditions have been submitted elsewhere. Experimental data were acquired and processed using the MassLynx v4.1 software package.

### Data analysis

Data were analyzed with IBM SPSS 19.0 (Statistical Package 2010, Chicago, IL), using Student’s t-test or one-way ANOVA followed by Dunnett’s multiple comparison test. Data are presented as the mean ± sem of 2–3 independent experiments, unless otherwise stated. Significance was set at P < 0.05. Analysis of the qRT-PCR data, which was normally distributed (Levene’s test), was performed using the ΔΔCt method. Differences among the control and treated groups were analyzed by Student’s t-test.

On the other hand, the autoscaled concentrations of neurotransmitters were represented in a heatmap plot with dendograms showing their hierarchical clustering (clustergram). Clustergram performs a hierarchical clustering analysis (HCA) of values and displays then heat map, with row and column dendrograms of the clustering. In our case, the rows in the input matrix were the neurotransmitter concentrations and columns, the samples. This heat map of the metabolite peak areas was calculated using the clustergram function in PLS Toolbox 7.3.1 (Eigenvector Research Inc., Wenatchee, WA, USA) from Matlab 2014a (Mathworks Inc. Natick, MA, USA)^62,63^.

### Data availability

The mass spectrometry proteomics data have been deposited to the ProteomeXchange Consortium via the PRIDE partner repository with the dataset identifier PXD007169. The authors declare that all other data supporting the findings of this study are available within the manuscript and its Supplementary Information files or are available from the corresponding author upon request.

## Electronic supplementary material

Supplementary Information

Supplementary Dataset 1

Supplementary Dataset 2

Supplementary Dataset 3

Supplementary Dataset 4
