# Supplementary material for: Modelling acrylamide acute neurotoxicity in zebrafish larvae
Source: Sci Rep. 2017 Oct 24;7:13952. doi: 10.1038/s41598-017-14460-3 (PMC5655329; doi:10.1038/s41598-017-14460-3)
Supplement: Supplementary file 1 — Supplementary Information [file 41598_2017_14460_MOESM1_ESM.pdf]

Supplementary Information

Modeling acrylamide acute neurotoxicity in zebrafish larvae

Eva Prats, Cristian Gómez-Canela, Shani Ben-Lulu, Tamar Ziv, Francesc Padrós, Daniel Tornero, Natàlia Garcia-Reyero, Romà Tauler, Arie Admon, Demetrio Raldúa[1]

Title of file for HTML: Supplementary Information
Description: Supplementary Methods, Supplementary Figures, Supplementary Tables, Supplementary References.

Title of file for HTML: Supplementary Dataset 1
Description: **List of all the zebrafish proteins identified by µLC-MS/MS in at least 3 of the samples and with at least 2 different tryptic peptides in the pools of control (PCN) and ACR-treated (PACR) larvae.**

Title of file for HTML: Supplementary Dataset 2
Description: **List of all the zebrafish proteins differentially expressed in the control and ACR-treated zebrafish larvae.**

Title of file for HTML: Supplementary Dataset 3
Description: **List of zebrafish proteins involved in the synaptic vesicle cycle identified by µLC-MS/MS in at least 3 of the samples and with at least 2 different tryptic peptides in the pools of control (PCN) and ACR-treated (PACR) larvae.**

Title of file for HTML: Supplementary Dataset 4
Description: **List of all the zebrafish proteins with covalent modifications on specific cysteine residues in at least 3 of the ACR-pools analyzed.**

Supplementary Information

Supplementary Methods

Stability of ACR in water determination

Acrylamide was spiked at 0, 0.5, 1.0 and 2.0 mM, and the obtained solutions were maintained in the same conditions used for the zebrafish larvae (28.5ºC and 12L:12D photoperiod). Aliquots were analyzed at time 0, 2, 6, 24, 48, 60, and 72 h. Analyses were carried out by direct sample injection using an liquid chromatography connected to a triple quadrupole detector (Xevo TQD, Waters, USA; LC–MS/MS). Data were acquired and processed using MassLynx 4.1 software package. A Synergi Polar-RP 80 Å column (250mm x 4.6 mm ID, particle size 4 µM, Phenomenex, Torrance, USA) was used. The mobile phase composition consisted of binary mixtures with 0.1% of formic acid in water (A) and 0.1% formic acid in acetonitrile (B). The initial mobile phase composition was 80 % A and 20 % B (5 min), to 70 % B in 7 min and to 100 % B in 2 min. These conditions were held for 1 min, and then, the initial conditions were regained in 2 min with a total run time of 12 min. The flow rate was set at 0.4 mL min-1. The flow rate was set at 0.4 mL/min. To optimize ionization and to establish mass spectral features, monomeric acrylamide were first analyzed by flow injection analysis (FIA) in LC-MS in a positive ion ESI mode. Full-scan data acquisition was performed by scanning from m/z 100 to 600. A profile mode was used to determine fragmentation patterns and sensitivity, using a scan time of 2s with a step size of 0.1 u and a pause between each scan of 2 ms. Optimized parameters were source temperature (from 125 to 150 ºC), cone

voltage (from 5 to 50 V) and collision energy (from 5 to 50 eV). Other parameters used are shown in Table S1.

Concentration-response analysis for lethality

LC50 values were obtained by fitting responses relative to control treatments (R) to the nonlinear allosteric decay regression model depicted in Eq. (1):

$$R(Ci) = \frac{1}{1 + \dfrac{C_i k}{\mathrm{EC/LC50}k}}$$

where

$R(C_i)$ proportional biological response at concentration C_i relative to controls

C_i concentration of the toxic substance (i)

LC50 the half saturation constant (concentration of ACR that caused 50% mortality of larvae)

k decay index

The allosteric decay model was selected to fit the obtained data because it can describe nonlinear type responses. Model accuracy was assessed using the adjusted coefficient of determination (r^2) and analysing residual distribution. The significance of the entire regression and regression coefficients were determined by analysis of variance (ANOVA) and Student's t-test, respectively.

All analyses were conducted using statistical analysis software (IBM SPSS 19.0 and SigmaPlot 11.0, 2008, Systat Software Inc.).

Behavioral analysis

Basal locomotor activity (BLA) and visual motor response (VMR) of 8 dpf zebrafish larvae were analyzed essentially as described by Faria et al. (2015)[1]. A minimum of 77 larvae were exposed per condition in at least two independent experiments. At the end of the exposure period, control and treated larvae were placed in 48-well microplates (NuncTM) containing 1 mL of experimental solution per well (internal diameter of 12 mm, flat bottom). The plate was then transferred into a behavioral testing chamber equipped with a temperature control unit (DanioVision, Noldus Information Technology, Leesburg, VA). Larvae were acclimated in the dark for 1 h before video recording. The video tracking conditions used consisted on 50 min cycle including a 20 min dark period followed by a 10 min light period and then a second cycle of 20 min of darkness. The position of each individual larva was recorded using an IR digital video camera Basler acA1300-60gm (Basler Inc., Exton, PA) and an EthoVision XT 9 video tracking system (Noldus Information Technology, Leesburg, VA). A dynamic subtraction method was applied, using a sampling rate of 60 images/s, dark contrast 20-250, current frame weight 1, subject-size 2-125000, and no subject contour dilatation. A minimum distance input with a filter of 10% of the total larva body, equivalent to 0.4 mm, was used to remove background noise. All measurements occurred in the afternoon between 1:00 and 6:00 pm, the optimal time interval for the stability of the basal activity. Tracks were analyzed for velocity (mm s-1) and total distance moved (cm) calculated for each dark or

light period. All microplates were analyzed at 28 ± 0.5°C with same detection and acquisition settings.

For the touch-evoked escape response, startle responses were evoked in 8 dpf control and ACR-exposed larvae (20-27 larvae per condition from three independent experiments) by a light-touch stimulus applied to the rostral head skin using a glass capillary injection needle. Video recordings were initiated 2 min after moving the larva to the testing arena to allow sufficient time for locomotor activity to stabilize. All video recordings were made with a high-speed Photron Fastcam MinI UX100 camera (Photron USA Inc., San Diego, CA, USA) at 896 × 896 pixel resolution using a Sigma 105 mm F2.8 EX DG Macro OS lens at 1000 frames per second. Individual larvae were tested in 6 cm Petri dishes. The plate was illuminated from below by a white LED back light LFL-100Sw2-IU with intensity control unit (CCS, Sint-Pieters-Leeuw, Belgium). The light intensity in the testing platform was measured using an ILT1400 radiometer (International Light Technologies Inc., Peabody, MA, USA) and adjusted to 300 μW/cm2. All behavioral measurements were made using the Flote software package. Briefly, this software performs tracking of the filmed larva and then performs an automated analysis of its body curvature to extract kinematic details of swimming movements. The curvature of the body was calculated for each frame and plotted over time using Microsoft Excel (Microsoft Corporation, Redmon, WA, USA).

Histopathological analysis

Larvae were fixed in 10% phosphate-buffered formalin (pH 7.2) at room temperature and subsequently dehydrated in a graded ethanol series (30, 50,

70, 90, 96 and 100%) for 30 min each. Larvae were progressively embedded in infiltration solution (Technovit® 7100, Kulzer) using absolute ethanol (1:3, 1:1 and 3:1) for 1 h in each step and finally soaked in 100% infiltration solution overnight at room temperature. The specimens were placed in Teflon block moulding cups (Kulzer), and the remaining infiltration solution was removed using a pipette. New infiltration solution with hardener II (15:1) was added to the cups and placed in an incubator (37°C). After polymerization, hardened methacrylate blocks were glued to plastic holders using Technovit® 3040 (Kulzer) and removed from the cups. Blocks were sectioned with tungsten knives in a rotary microtome (Reichert-Jung). Sections (2 μm thick) were floated on a distilled water bath at room temperature, picked up on clean glass slides, dried at 40°C and immersed in an aqueous solution of 1% Toluidine Blue for 2 min and rinsed in tap water until the desired blue intensity and contrast with the background. Finally, sections were dried at 37°C and mounted using DPX.

Supplementary Figures

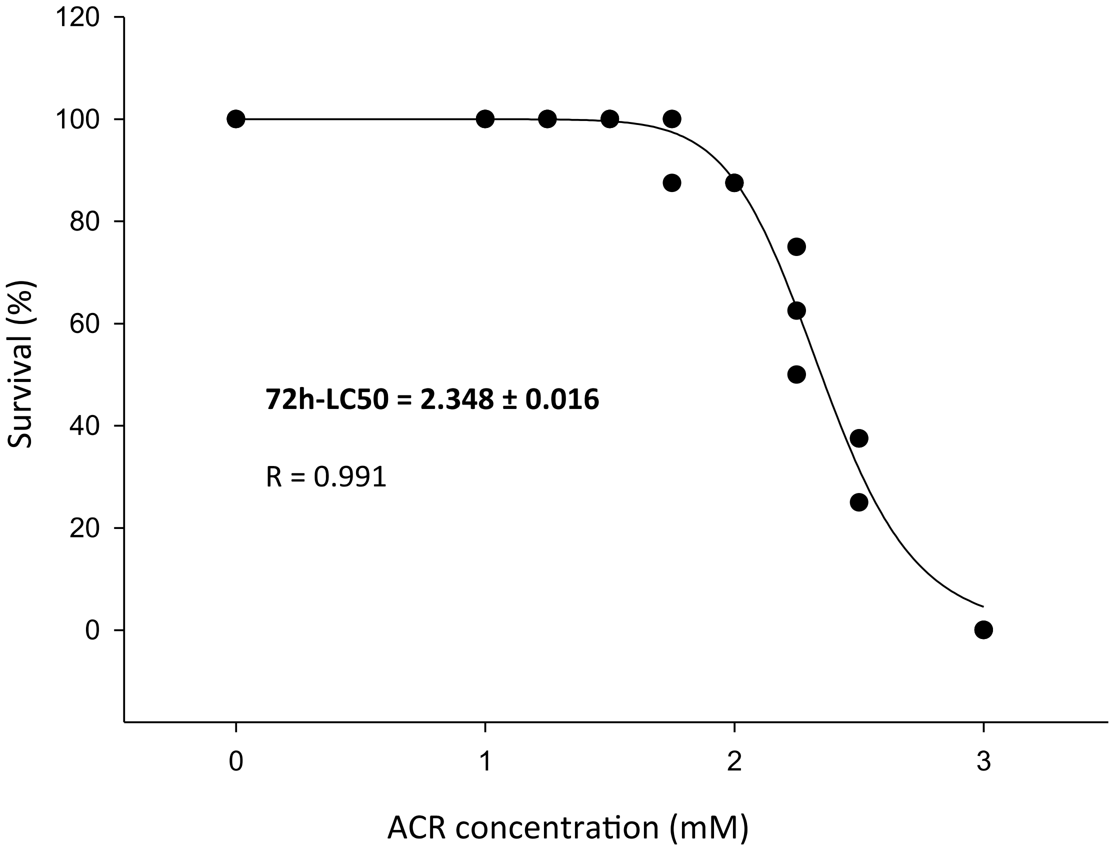

Supplementary Figure S1: Concentration-response analysis of the effect of ACR on the survival of zebrafish larvae. Regression parameters according to Eq.1 of fitted models to mortality responses were: LC50: 2.35 mM ACR; / s.e.m: 0.02/ $P < 0.0001$/ r^2: 0.9813/ n= 237. Abbreviations: ACR, acrylamide.

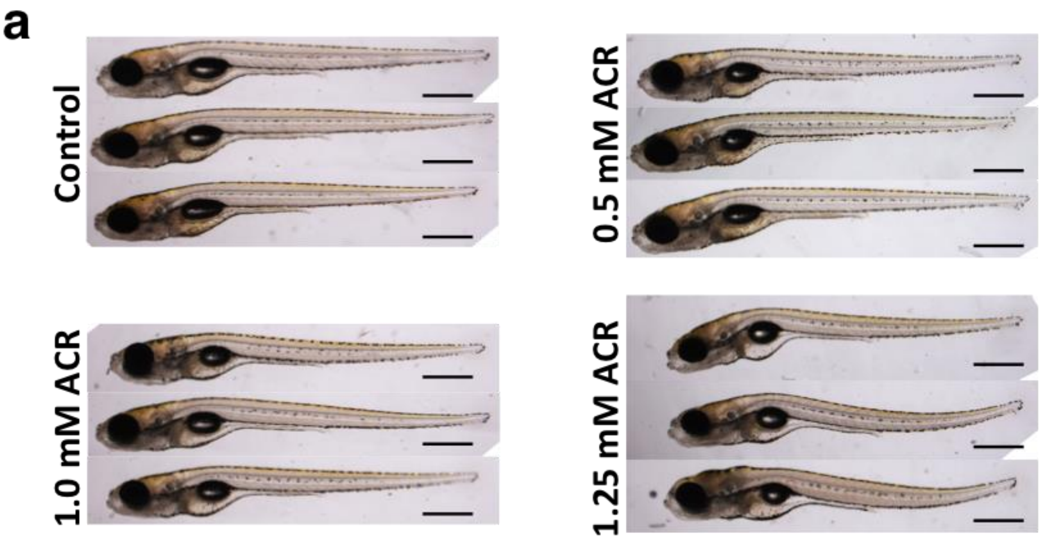

b

Total body length (cm)

**Control** (n=30)	3.81 ± 0.03	
**0.5 mM ACR** (n=32)	3.83 ± 0.04	NS (vs Control)
**1.0 mM ACR** (n=28)	3.78 ± 0.03	NS (vs Control)

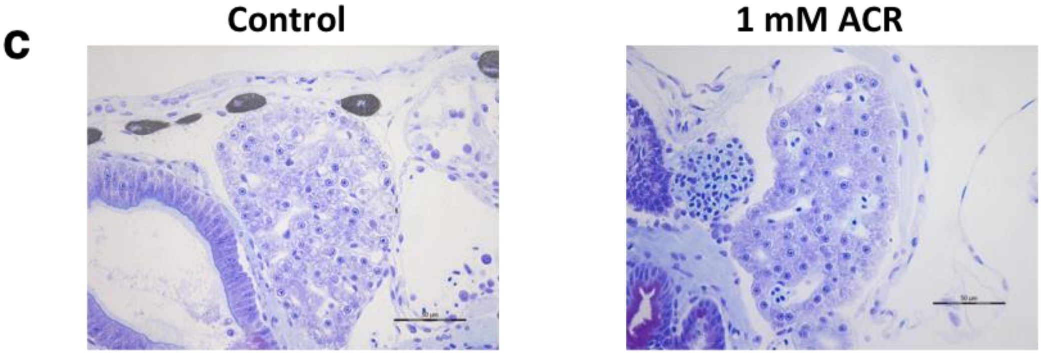

Supplementary Figure S2: Determination of MTC for ACR systemic toxicity. (**a**) Gross morphology assessment shows that whereas 1.25 mM ACR is the LOEC, 1 mM ACR is the NOEC for this endpoint. (**b**) Morphometric analysis shows that 0.5-1.0 mM ACR has no effect on total length of the larvae. Statistical analysis was performed using one-way ANOVA with Dunnett's multiple comparison test. Results represent mean ± sem; NS: not significant (**c**) Exposure to 1 mM ACR has no effect on liver morphology. Scale bar: (**a**) 500 μm; (**c**) 50 μm.

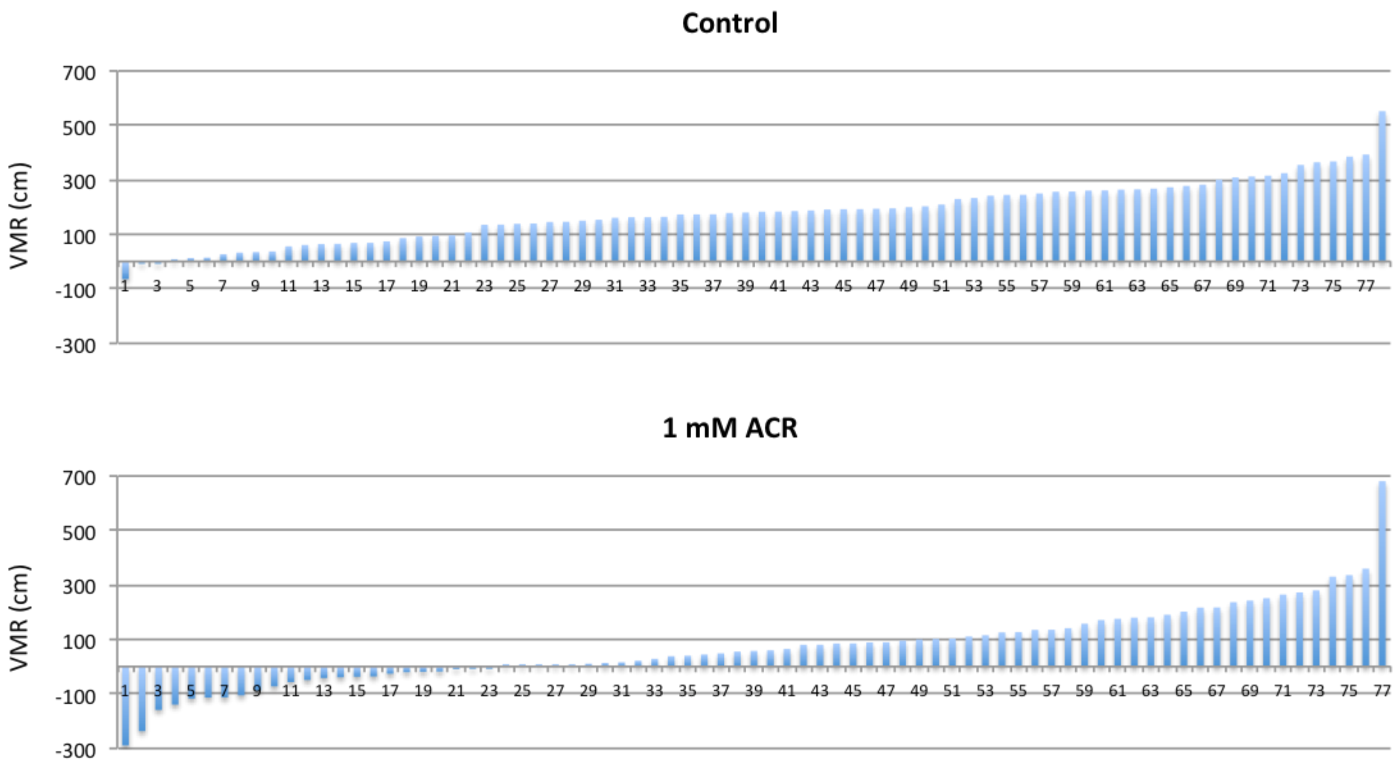

Supplementary Figure S3: Distribution of VMR values in 77 control and ACR-exposed larvae. ACR treatment results in a shift to the left in the distribution of VMR value. VMR was defines as the difference between the distance moved by the larvae during the first 2 min in the second period in dark (min 24-26) and the last 2 min of the period in light (min 22-24).

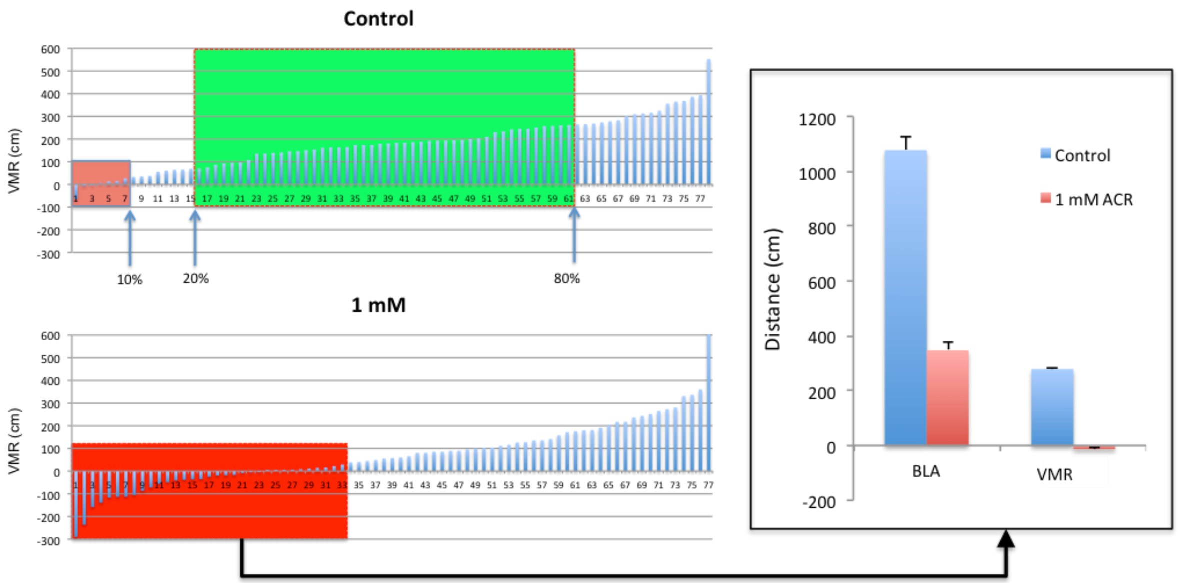

Supplementary Figure S4: Criteria for selection samples. First of all, all the larvae were screened for visual motor response (VMR) using a protocol consisting in 14 min dark/ 10 min light/ 4 min dark. VMR was defined as the difference between the distance moved by the larvae during the first 2 min in the second period in dark (min 24-26) and the last 2 min of the period in light (min 22-24). Larvae control with VMR values between 20th and 80th percentiles (green box) were selected as “control larvae” for neurotoxicity assessment. ACR-treated larvae with VMR values below the 10th percentile of the controls (red box) were selected for neurotoxicity assessment. Values for the basal locomotor activity (BLA; defined here as the distance moved during the 14 min dark period of the screening) and VMR of the selected control and ACR-treated larvae after screening all the exposed larvae in a representative experiment.

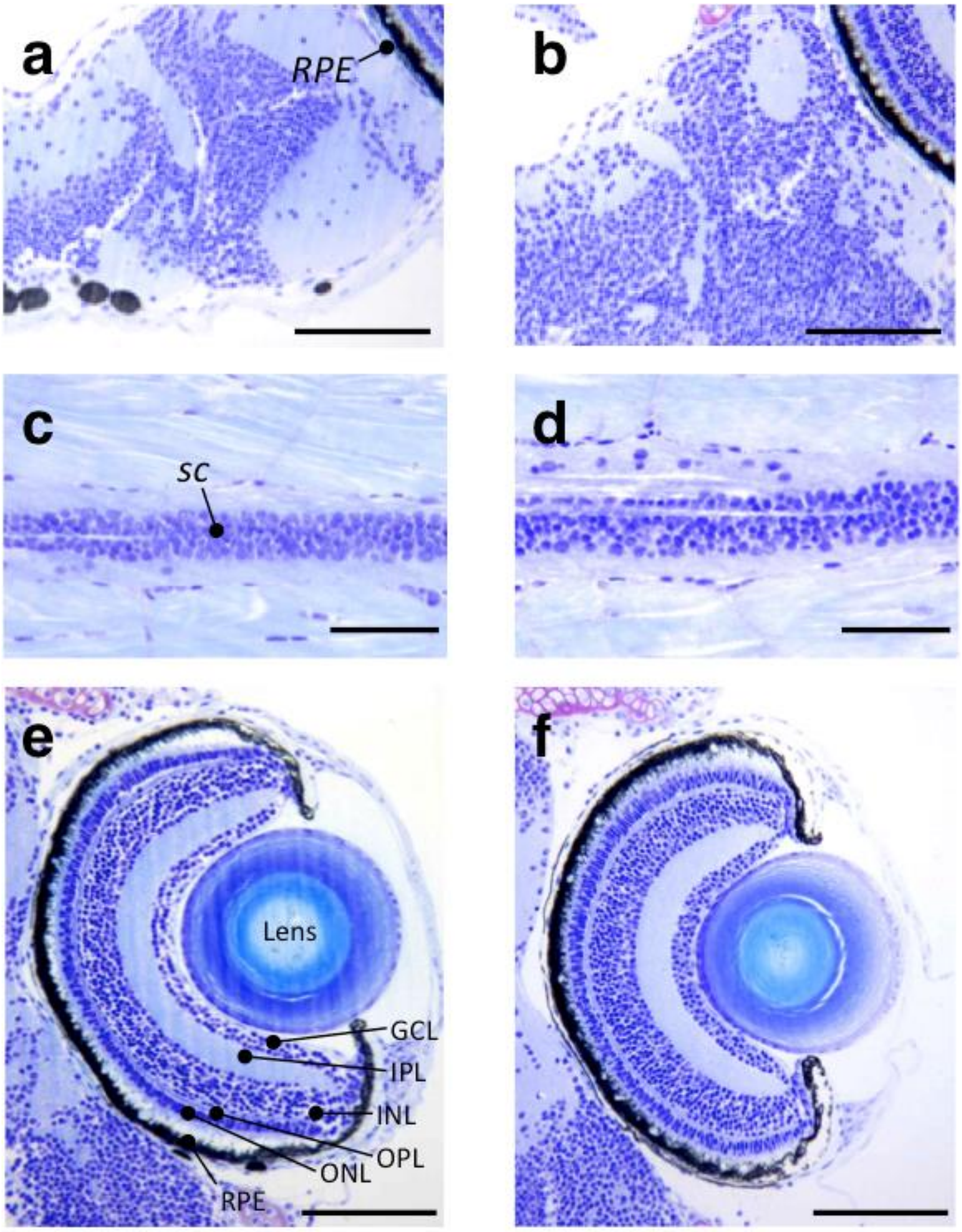

Supplementary Figure S5. Histopathological analysis of control and 1 mM ACR exposed larvae. Representative semithin sections of the brain (**a,b**), spinal cord (**c,d**) and the retina (**e,f**) of control (**a,c,e**) and ACR-treated (**b,d,f**) zebrafish larvae. No histopathological changes were identified using this methodology. Abbreviations: *GCL*, ganglion cell layer; *INL*, inner nuclear layer; *IPL*, inner plexiform layer; *ONL*, outer nuclear layer; *OPL*; outer plexiform layer; *RPE*, retinal pigment epithelium; *sc*, spinal cord. Scale bar: (**a,b,e,f**) 100 µm, (**c,d**) 50 µm.

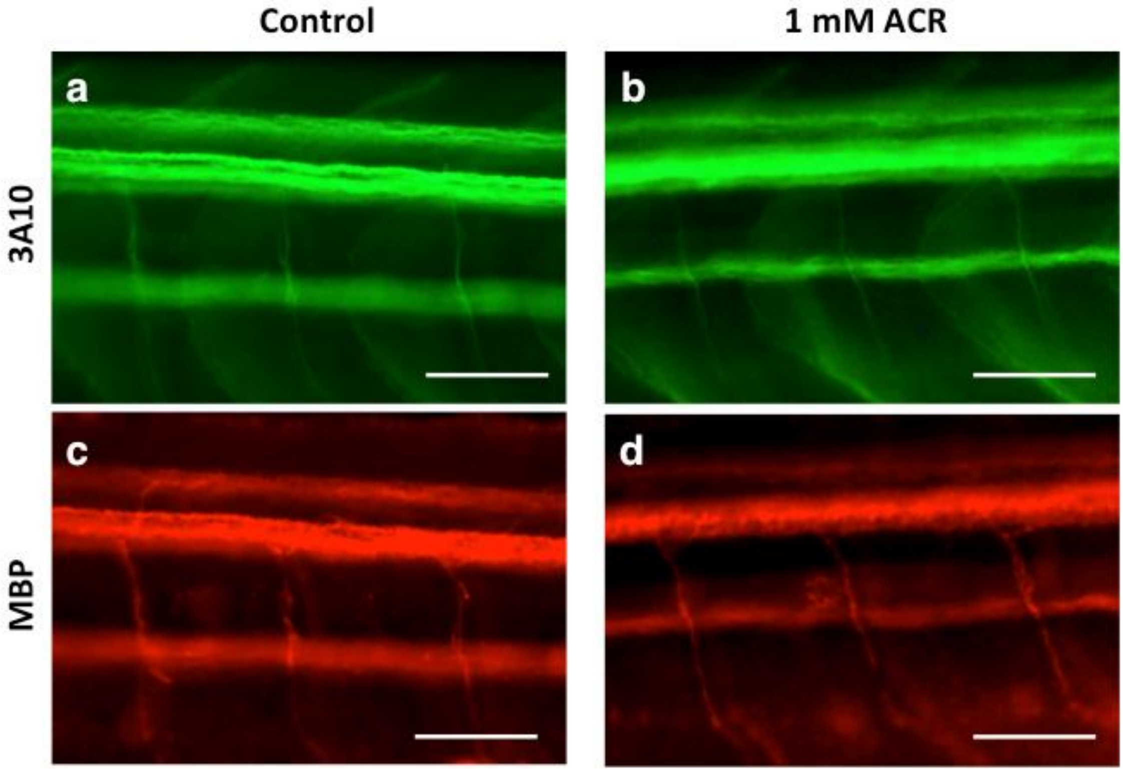

Supplementary Figure S6. Axonal tracts and myelin sheets are not primary targets for acrylamide neurotoxicity in zebrafish larvae. Detail of the trunk, in lateral view, of control (**a,c**) and ACR-treated larvae (**b,d**) after double whole-mount immunofluorescence with 3A10 antibody labelling axonal tracts and MBP antibody labelling the myelin sheets. No clear differences in both structures were evident between the two groups. Scale bar: 100 μm.

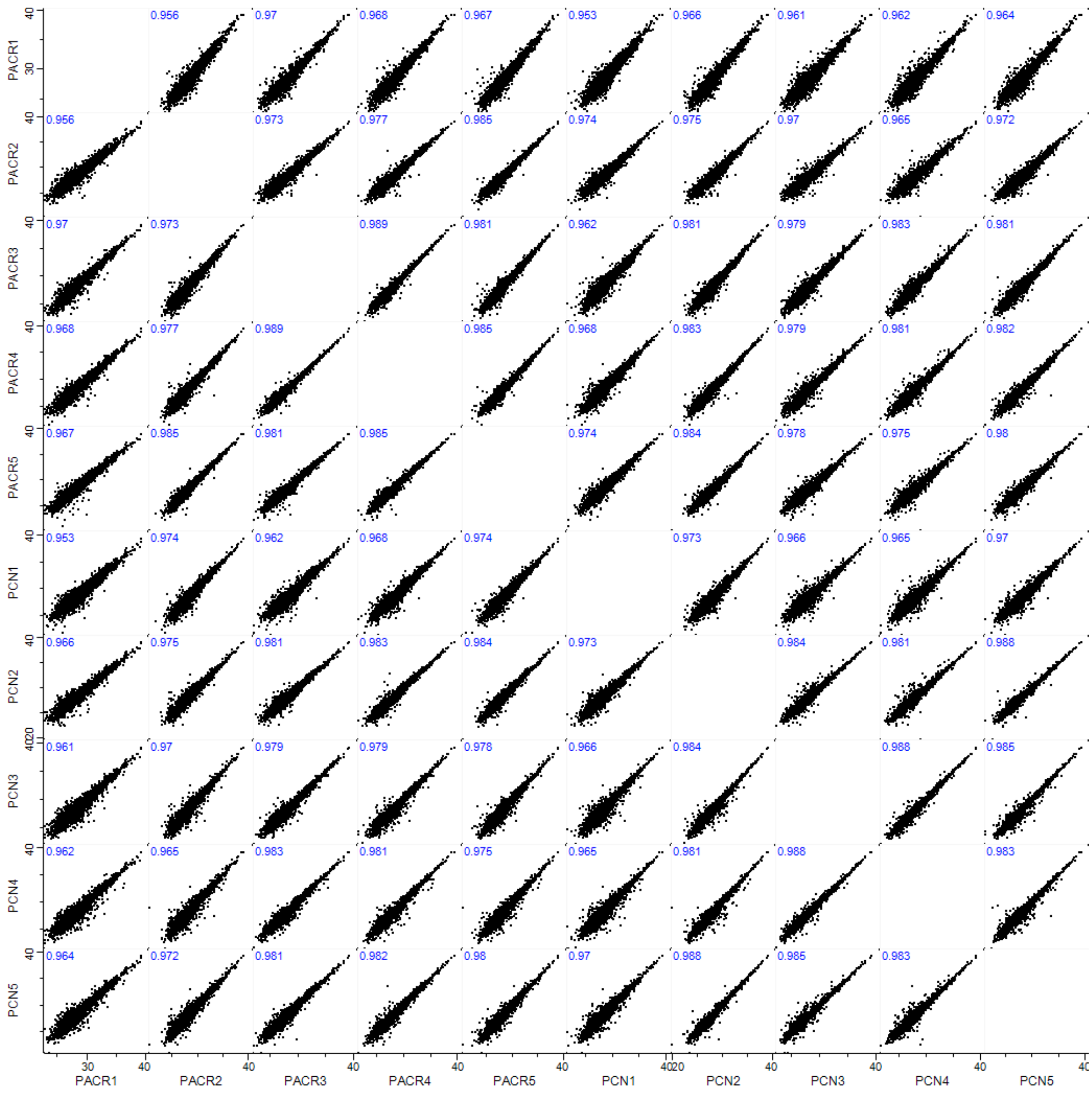

Supplementary Figure S7. Scatter plot indicating the LC-MS-MS signal intensities of the different protein in the treated and in the untreated fish. The numbers in each box indicate the Pearson correlations between two samples. The very high correlations observed here indicate that the proteome analyses were very reproducible. Signal intensities are on a Log2 scale and each dot represents another protein.

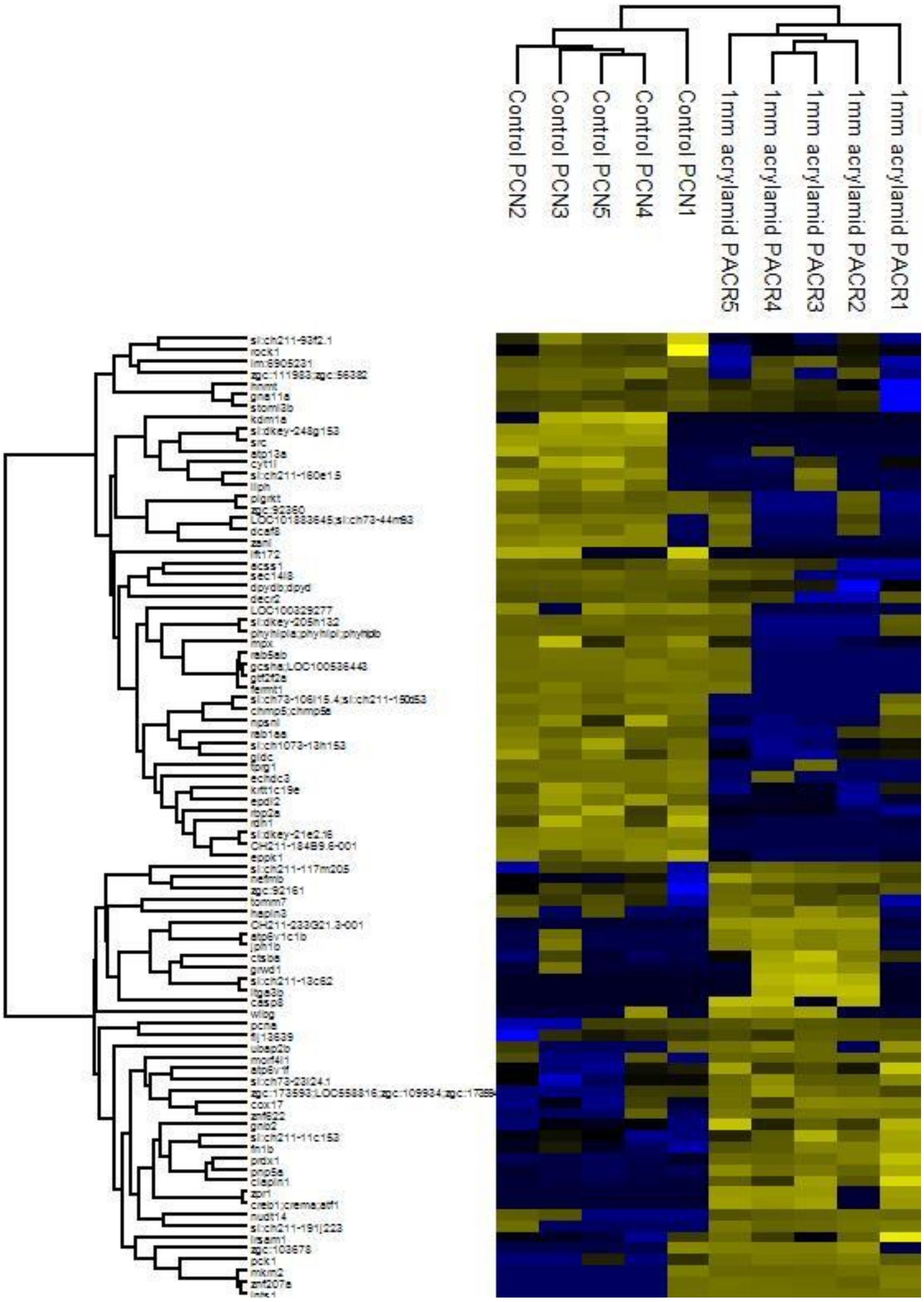

Supplementary Figure S8. The effect of acrylamide treatment on the levels of the zebrafish proteins. The heat-map depicts the label free quantification (LFQ) as calculated by the MaxQuant software tool using the LC-MS-MS signal intensities listed in Supplementary Table S5. The data is Z-scored and displays on the proteins that were observed to change significantly in at least three biological replica using the Perseus software.

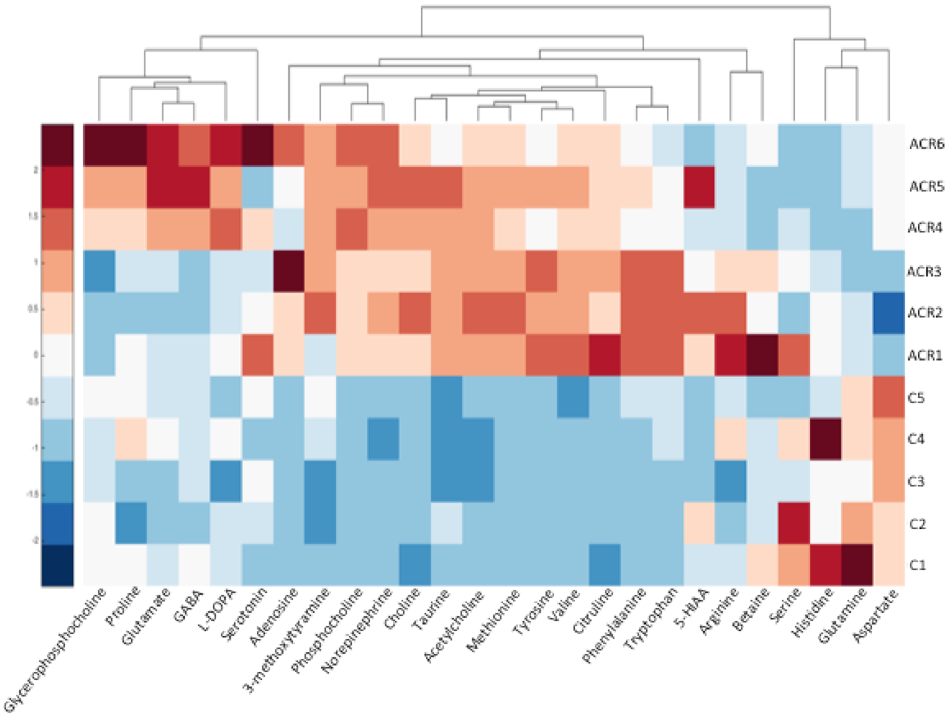

Supplementary Figure S9. Heat map of the ACR-effect on the neurochemicals profile in zebrafish. Neurochemicals are represented in the columns, and the samples [controls (C1-C5) and ACR-treated larvae (ACR1-ACR6)] in the rows.

Supplementary Tables

Supplementary Table S1: HPLC–MS/MS conditions used to determine acrylamide.

**Column**	Synergy Polar-RP 80 Å
**Chromatographic parameters**	
Injection volume	10 µL
Flow rate	400 µL min^{-1}
Mobile phase A	H_2O with 0.1% HCOOH
Mobile phase B	ACN with 0.1% HCOOH
Acquisition time	12 min
**Source parameters**	
Scan range	*m/z* 100-600
Capillary	3.5 kV
Extractor	3 V
Polarity	Positive (ESI+)
Source Temperature	150 ºC
Desolvation Temperature	350 ºC
Cone gas flow	1 L h^{-1}
Desolvation gas flow	550 L h^{-1}
Collision gas flow	0.19 mL min^{-1}
**Analyzer parameters**	
LM1 Resolution	15.00
HM1 Resolution	15.00
Ion energy 1	0.50
MS Mode Entrance	10.00
MS Mode Collision Energy	3.00
MS Mode Exit	10.00
LM2 Resolution	15.00
HM2 Resolution	15.00
Ion energy 1	0.50
MS Mode Entrance	1.00
MS Mode Collision Energy	20.00
MS Mode Exit	0.50
Gain	1.00
Multiplier	-651.31

SupplementaryTable S2 Primers used for qPCR, including ZFIN and GenBank accession numbers.

Gene	ZFIN	GenBank	Gene description		Sequence	Amplicon
gfap	ZDB-GENE-990914-3	NM_131373	glial fibrillary acidic protein	FW	5'-GGATGCAGCCAATCGTAAT	97 bp
				RV	5'-TTCCAGGTCACAGGTCAG	
mbp	ZDB-GENE-030128-2	AY860977	myelin basic protein a	FW	5'-AATCAGCAGGTTCTTCGGAGGAGA	102 bp
				RV	5'-AAGAAATGCACGACAGGGTTGACG	
gap43a	ZDB-GENE-990415-87	NM_131341.1	growth associated protein 43	FW	5'-CAGCCGACGTGCCTGAA	71 bp
				RV	5'-GGATTCCTCAGCAGCGTCTG	
syn2a	ZDB-GENE-040718-341	NM_001002597	synapsin IIa	FW	5'-GTGACCATGCCAGCATTTC	80 bp
				RV	5'-TGGTTCTCCACTTTCACCTT	
α-tub	ZDB-GENE-030822-1	NM_194388.2	tubulin, alpha 1b	FW	5'-AATCACCAATGCTTGCTTCGAGCC	117 bp
				RV	5'-TTCACGTCTTTGGGTACCACG	
nsfa	ZDB-GENE-030616-37	NM_001044328.1	N-ethylmaleimide-sensitive factor a	FW	5'-CGCGGCTTCTTCGAGTAACA	134 bp
				RV	5'-GAAGTGTGATCTCCGTCAGGTT	
syt1a	ZDB-GENE-040718-165	NM_001327829	synaptotagmin Ia	FW	5'-AAAGGGAAGAGACGGCTGTG	130 bp
				RV	5'-GGAGCCAGGCAGAAGCTTTA	
stxbp1b	ZDB-GENE-060531-166	NM_001089376.1	syntaxin binding protein 1b	FW	5'-ACGCTGAAAGAGTACCCAGC	118 bp
				RV	5'-CTCCCAAAGTGGGGTCATCC	
vamp2	ZDB-GENE-030131-8225	NM_200005.1	vesicle-associated membrane protein 2	FW	5'-CGCAACATTCCTACCCCACT	99 bp
				RV	5'-GTGAGAAGTCGTTGCTCCCA	
opn1lw1	ZDB-GENE-990604-41	NM_001313715.1	opsin 1 (cone pigments), long-wave-sensitive, 1	FW	5'-TGGAGCAGATACTGGCCTCAT	71 bp
				RV	5'-GGGTCCTCGCTTCCACTGA	
opn1mw1	ZDB-GENE-990604-42	NM_131253.2	opsin 1 (cone pigments), medium -wave-sensitive, 1	FW	5'- AGCCGATGGGTCTGAGGTTAA	81 bp
				RV	5'- TGCCTCCACCTAACAGTGAAGAA	

Supplementary Table S3: Stability of ACR in fish water under the experimental conditions

Nominal concetration (mM)	Time (h)	Measured concentration (mM)	Measured concentration (mean ± SE)
0.50	0	0.481<br>0.538<br>0.481	0.50 ± 0.02
	6	0.488<br>0.438<br>0.443	0.46 ± 0.02
	24	0.385<br>0.497<br>0.450	0.44 ± 0.03
	48	0.381<br>0.475<br>0.415	0.42 ± 0.03
	60	0.439<br>0.406<br>0.481	0.44 ± 0.02
	72	0.422<br>0.496<br>0.558	0.49 ± 0.04
1.00	0	1.226<br>0.994<br>1.132	1.12 ± 0.07
	6	1.019<br>0.899<br>0.991	0.97 ± 0.04
	24	0.992<br>1.054<br>1.002	1.02 ± 0.02
	48	0.985<br>0.900<br>0.860	0.92 ± 0.04
	60	0.843<br>0.923<br>0.982	0.92 ± 0.04
	72	1.035<br>1.081<br>1.248	1.12 ± 0.06
2.00	0	1.828<br>1.902<br>1.907	1.88 ± 0.03
	6	2.007<br>1.883<br>2.083	1.99 ± 0.06
	24	1.874<br>2.132<br>1.895	1.97 ± 0.08
	48	1.687<br>1.766<br>1.514	1.66 ± 0.07
	60	1.747<br>1.859<br>1.736	1.78 ± 0.04
	72	2.220<br>2.145<br>2.184	2.18 ± 0.02

Supplementary Table S4. Levels of the main neurotransmitters, precursors and degradation products in 8 dpf zebrafish larvae control and exposed to 1 mM ACR for 72 h.

	Control	1 mM ACR	*P* value
**Choline (ng/larva)**	10.67 ± 0.43	23.44 ± 0.9	8.009×10^{-7}
**Acetylcholine (ng/larva)**	0.69 ± 0.06	2.30 ± 0.09	2.282×10^{-7}
**Tryprophan (ng/larva)**	1.95 ± 0.13	3.70 ± 0.45	7.117×10^{-3}
**Serotonin (pg/larva)**	20.38 ± 3.27	33.85 ± 6.99	0.137
**5-HIAA (pg/larva)**	57.97 ± 4.22	74.99 ± 6.64	0.070
**Phenylalanine (ng/larva)**	2.09 ± 0.19	6.91 ± 0.85	6.952×10^{-4}
**Tyrosine (ng/larva)**	1.27 ± 0.07	4.20 ± 0.39	9.223×10^{-5}
**L-DOPA (ng/larva)**	16.67 ± 0.82	21.00 ± 1.46	0.038
**3-Methoxytyramine (pg/larva)**	64.99 ± 11.79	133.89 ± 9.13	1.123×10^{-3}
**Norepinephrine (pg/larva)**	21.18 ± 0.64	46.20 ± 2.89	3.020×10^{-5}
**Glutamine (ng/larva)**	78.90 ± 7.96	48.65 ± 1.50	2.663×10^{-3}
**Glutamate (ng/larva)**	48.05 ± 1.94	57.46 ± 4.91	0.134
**GABA (ng/larva)**	10.45 ± 0.45	13.10 ± 1.58	0.173

Supplementary References

1 Faria, M. *et al.* Zebrafish models for human acute organophosphorus poisoning. *Sci Rep.* **5**, 15591 (2015).
